# The not-always-uniquely-predictive power of an evolutionary approach to understanding our not-so-computational nature

**DOI:** 10.3389/fpsyg.2015.00419

**Published:** 2015-04-21

**Authors:** Gert Stulp, Thomas V. Pollet, Louise Barrett

**Affiliations:** ^1^Department of Population Health, London School of Hygiene & Tropical MedicineLondon, UK; ^2^Department of Social and Organizational Psychology, VU University AmsterdamAmsterdam, Netherlands; ^3^Department of Psychology, University of LethbridgeLethbridge, AB, Canada

**Keywords:** evolutionary psychology, cognition, cognitive integration, modules, extended mind

## Introduction

We thank Klasios (2014)^1^ and Stephen et al. (2014)^2^ for their commentaries on our paper (Barrett et al., 2014)^3^. Criticisms like these can only help to improve the quality of arguments offered on both sides. Both Klasios's and Stephen et al.'s commentaries generate misconceptions, however, about the aim of our article and our stated position. Before we respond more generally to their arguments, we want to correct these mistaken impressions.

First, Klasios states that we misinterpret the EP notion of computation. This is simply false. We do not argue that EP posits a physical architecture. Our characterization of the EP view (p. 3) is that it “relies heavily on analogies to computational algorithms, functions, inputs, and outputs” and that its research strategy “involves … hypothesizing the kinds of algorithmic “design features” that any psychological adaptation would require in order to solve such a problem.” The notion of a physical architecture was raised in response to Robert Kurzban's implied suggestion that psychological adaptations are analogous to morphological (i.e., physical) adaptations, and can be reverse engineered in the same way. We disputed Kurzban's argument precisely because there are no grounds for positing a particular kind of physical architecture that could serve to support such an analogy (see also Peters, 2013).

Second, Stephen et al. (2014) present our argument as stating that the primary contribution of an evolutionary approach to psychology is the idea of massive modularity when, in fact, we stated merely that the modularity of evolved adaptations is the primary *distinction* between EP and standard computational theories. We do not consider modularity to be the primary contribution of an evolutionary approach to human behavior, as should be clear from our previous work (e.g., Barrett et al., 2001; Pollet et al., 2009; Stulp and Barrett, 2014), as well as our argument in the original paper.

Both these misconceptions perhaps arise because of a failure to appreciate that we were addressing the specific question posed for this research topic: is EP the obvious alternative to standard computational approaches to the mind? Our answer was that one evolutionary approach to psychology (i.e., the “Santa Barbara School” of Evolutionary Psychology, which we refer to as EP throughout this piece) was not an alternative approach, nor could it be, precisely because it *is* a computational theory. As such, it could only be distinguished from other cognitive approaches via the manner in which it applied evolutionary thinking. A large part of our paper was then devoted to why we felt the particular evolutionary approach adopted—namely, modular psychological adaptation—fell short. Other evolutionary approaches are not vulnerable to this criticism, and so we did not include these in our analysis. Thus, nowhere do we dispute Stephen et al.'s main point that evolutionary theory is useful for explaining human behavior and that, using Tinbergen's integrative approach, one can generate unique predictions (see also Barrett and Stulp, 2013).

## The not-always-so-uniquely predictive ability of evolutionary theory

We do want to add, however, that evolutionary theory may not always be as uniquely predictive as Stephen et al.'s examples suggest. With respect to disgust, we read (p. 2): “the principal driver behind studying this emotion's relationship with the immune system was based upon the idea that disgust functions to aid disease avoidance (Stevenson et al., 2011)” and that such “avenues of enquiry would not have been envisaged” without evolutionary theorizing. Their confidence in the latter statement is perhaps misplaced, however, as Stevenson et al.'s (2011) research drew explicitly on earlier findings by Bosch et al. (2001) that were obtained without any reference to either disgust or evolutionary theory (as acknowledged by Stevenson et al., p. 900). At best, then, we might say that, in this case, the functional explanation represents a plausible *post-hoc* account and further clarification of an already established phenomenon, rather than that the functional perspective made unique predictions that allowed the phenomenon to be identified in the first place.

Similarly, in their second example, Stephen et al. predict that species with trichromatic vision (i.e., the ability to distinguish red from green) will make use of red coloration as cues and signals more than dichromats. Again, the prediction that animals unable to see red will not make use of red coloration, whereas those animals that can see red potentially might do so, is in itself not a very strong evolutionary prediction, given that one could just as easily formulate such a prediction solely on an understanding of extant species' visual systems. It is, however, important to note that we are not denying the importance of evolutionary theory. Our point is simply that we should not overstate its power to generate unique predictions and empirical findings that would otherwise not occur. Similarly, most findings in evolutionary psychology are, as the authors of many of these articles themselves note, consistent with evolutionary predictions, but do not rule out other potential explanations.

## Ultimately proximate?

Stephen et al.'s second point is that we have confused proximate and ultimate levels of explanation. Specifically, they state that “Barrett et al.'s (2014) conception of e-cognition as an alternative to evolutionary approaches to cognition and behavior mischaracterizes e-cognition as an ultimate explanatory framework, when it should properly be considered proximal” (p. 2). We believe this criticism is unwarranted for several reasons. First, we raised E-cognition as an alternative to the standard cognitivist, computational approach to psychology, and *not* to a functional evolutionary approach. We do not dispute that phylogenetic and functional levels of explanation can provide additional “explanatory value above standard computation models” (p. 3). Our actual argument was that E-cognition, and cognitive integration in particular, could fill some of the gaps left open by current information-processing approaches, and we said nothing to suggest that this should occur to the exclusion of evolutionary theory. Rather, our point was that, to take Stephen et al.'s example, seeking answers to how and why humans can program things like iPhones (and obviously conceive of and manufacture them in the first place) seems crucial to achieving a “fuller understanding of cognition and behavior” (p. 3) than we currently possess. Stephen et al. must surely agree that such a full understanding goes well beyond the phylogenetic and functional levels of explanation, and our closing plea for explanatory pluralism was made precisely for this reason. Klasios, while (surprisingly) using this point against us, actually agrees on this when he states that “[a]t a pragmatic level, different research programs will simply find it profitable to have differing explanatory focuses and emphases” (p. 1).

Second, nowhere did we “mischaracterize” E-cognition as an ultimate framework, in just the same way that no one argues that information-processing theories represent an ultimate framework. Both are guiding theories that take a particular stance on the nature of cognitive processes and, in that sense, both can be seen as more proximate than ultimate. Some E-cognition theories may stand on their own without referring to evolutionary theory, in the same way that most information-processing-theories similarly lack this explicit connection. That said, Stephen et al. perhaps jumped the gun by stating that E-cognition is a proximate and not an ultimate approach, without providing any reason as to why this is the case. Indeed, some forms of E-cognition are fundamentally evolutionary. For instance, certain aspects of the extended mind argument have been made in an explicitly evolutionary way, captured by Clark's (2005) “007 Principle” and Rowlands' (2003) “barking dog principle.” Both of these suggest that a thrifty evolutionary process will not build internal resources (especially expensive neural tissue) if the structure of the environment itself can be exploited in a way that can bear some of the cognitive burden. Distributed, extended cognition is thus the process by which internal resources are replaced or complemented by reliable external structures, with the idea that organisms that pursue this route will achieve higher fitness. This is supported by analogies from other species (for example, the manner in which the physics of a cricket's body automatically filters out extraneous sounds; a process that would otherwise need to be performed by neural tissue: Barrett, 2011) and so the extended mind also adopts the phylogenetic perspective for which Stephen et al. advocate.

## Openness to change rather than constancy as a constant

Klasios, in contrast, believes that our suggestion for E-cognition as an alternative to standard computational approaches stems from our flawed understanding of computation, and that “there is nothing within the theoretical approach of evolutionary psychology that in principle denies the existence of … ‘E-cognition’” (p. 1). We disagree strongly with this point, and tackle it in conjunction with Klasios's assessment that “our discussion of human nature is also problematic” (p. 1). The latter assertion seems to be based on a misreading of Wheeler and Clark (2008) statement that our extended cognitive architecture's “constancy lies mainly in *its continual openness to change*” (p. 3572, emphasis added). While we take “change” to be the key here, Klasios takes it to be “constancy,” arguing that EP recognizes “this underlying constancy and refers to it as our underlying ‘developmental programs’” (p. 1). Klasios' apparent misunderstanding of Wheeler and Clark's position leads nicely into a consideration the fundamental differences between E-cognition and computational theories.

It is important to note that we completely agree that some varieties of E-cognition can be seen as complementary to computational theories, given that they raise no objections to a rules-and-representations approach (as we explicitly addressed in our paper; p. 10). The EP position on psychological phenomena as adaptations, however, does not, in fact, gel very well with E-cognition, since the latter argues for the deep intertwining of brain, body and environment, whereas EP emphasizes a disjunction between these elements. EP's premise is that cognitive processes occur in the brain alone, and that our psychology is adapted to a past (environment) that in large part no longer exists, hence we are often mismatched to the modern world (e.g., Tooby and Cosmides, 1990). In this view, our psychological processes may often operate in opposition to the world around us, whereas the E-cognition view is that body and environment should be considered as integral parts of the cognitive system. We can see this even more clearly in Klasios's suggestion that EP deals only with a functional level of explanation that “abstracts away from instantiations in brain, body and the larger context in which they are embedded” (p. 1). In addition to the fact that the concept of an “abstracted adaptation” is entirely unclear to us, this position is fundamentally at odds with an E-cognition view which holds that no such abstraction is possible because cognitive processes are precisely a function of a brain embedded in a body embedded in an environment, all of which make crucial, often constitutive, contributions to those cognitive processes (e.g., Clark, 1997).

Klasios goes on to suggest that we misrepresent the EP view on human nature by neglecting Sperber's notion of epidemiological culture (see e.g., Sperber, 1996), which, according to Klasios, is equivalent to an E-cognition approach. Sperber's (1996) argument is, however, focused more strongly on how existing (evolved) psychological structures influence the kinds of cultural patterns produced (as captured in his notion of “cultural attractors”), with less emphasis placed on how culture actively alters our psychology. This is perhaps to be expected given that Sperber (1996) adheres to a standard EP view of psychological adaptations (modules) to past environments (also note that epidemiological or transmitted culture is given far less prominence in Tooby and Cosmides' conception of culture than Klasios suggests). Sperber's more recent position is that cultural phenomena “invade and inflate” our evolved mental modules, often resulting in “mismatches” between evolved function and current usage (Sperber and Hirschfeld, 2004), whereas cognitive integration argues that our psychology is never fixed but continually transformed as it incorporates various kinds of cultural artifacts (including the iPhone).

## Conclusion

Again, we would like to thank John Klasios and Ian Stephen and colleagues for engaging in this discussion and providing us with the opportunity to clarify our position. We hope our original paper and this reply continue to spark debate on our computational nature or lack thereof.

### Conflict of interest statement

The authors declare that the research was conducted in the absence of any commercial or financial relationships that could be construed as a potential conflict of interest.

## Footnotes

^1^http://journal.frontiersin.org/article/10.3389/fpsyg.2014.01348/full

^2^http://journal.frontiersin.org/article/10.3389/fpsyg.2014.01372/full

^3^http://journal.frontiersin.org/article/10.3389/fpsyg.2014.00867/full
